# Alkaline phosphatase activity in gingival crevicular fluid during orthodontic treatment with different extraction protocols for maxillary canines: a randomized controlled trial

**DOI:** 10.1038/s41405-026-00425-0

**Published:** 2026-05-02

**Authors:** Yahya Dakdouk, Kindah Sultan, Shadi Azzawi

**Affiliations:** 1 Department of Orthodontics, Faculty of Dentistry, University of Damascus, Damascus, Syria; 2 Department of Pediatric Dentistry, Faculty of Dentistry, University of Damascus, Damascus, Syria

**Keywords:** Fixed appliances, Periodontics

## Abstract

**Background:**

Alkaline phosphatase (ALP) activity in gingival crevicular fluid (GCF) reflects localized periodontal and alveolar bone remodeling during orthodontic tooth movement. Although ALP changes have been investigated during leveling and alignment or active orthodontic movement, the biological impact of tooth extraction timing relative to orthodontic force application remains insufficiently characterized.

**Objective:**

This randomized controlled trial aimed to compare ALP activity in gingival crevicular fluid between different extraction protocols in maxillary canines over a 6-week period. The primary outcome was the between-group difference in ALP activity across repeated time points.

**Methods:**

Sixty patients with moderate maxillary crowding were randomly allocated, using concealed allocation into three equal groups: Group A (leveling and alignment only with delayed extraction), Group B (extraction only), and Group C (simultaneous extraction with leveling and alignment). GCF samples were collected weekly from Mesial and Distal sites of maxillary canines from baseline to week six. ALP activity was quantified using a spectrophotometric method. Data distribution was assessed prior to analysis. Due to predominantly non-normal distributions, non-parametric tests were primarily applied, including Friedman’s test for intragroup comparisons and Kruskal–Wallis test for intergroup comparisons, with appropriate post-hoc analyses.

**Results:**

Distinct temporal patterns of ALP activity were observed across the three groups. Group B (extraction only) demonstrated the highest ALP activity during the early time points, particularly at weeks 1–3, while Group C showed intermediate responses and Group A exhibited consistently lower levels. At T1, significant intergroup differences were observed. At Mesial sites, Group C showed higher ALP activity than Group A (mean difference: 0.59 IU/L), followed by Group B (mean difference: 0.28 IU/L compared with Group A; *p* < 0.05). At Distal sites, Group B demonstrated the highest ALP activity compared to Group A (mean difference: 1.00 IU/L; *p* < 0.01), with Group C showing intermediate values compared with Group A (mean difference: 0.73 IU/L).

**Conclusions:**

Tooth extraction was associated with increased ALP activity in gingival crevicular fluid during the early phase of orthodontic treatment. Variations in ALP patterns between extraction protocols indicate that extraction timing modulates early biochemical responses in periodontal tissues.

## Introduction

Orthodontic tooth movement is a biologically complex process involving coordinated responses of the periodontal ligament (PDL) and alveolar bone, mediated through an interconnected cascade of cellular and molecular mechanisms [1, 2]. As a result of these localized biological responses, increasing attention has been directed toward the surrounding biological environment as a source of measurable indicators of orthodontic activity. In this context, gingival crevicular fluid (GCF) is widely regarded as a sensitive indicator of such events, as it reflects local cellular and enzymatic activities within the periodontium [3]. Among the various enzymes detected in GCF, alkaline phosphatase (ALP) has received particular attention as a well-established biological marker associated with osteoblastic activity and bone formation processes [4, 5]. ALP was selected as a primary biomarker in this study due to its well-documented association with osteoblastic activity and its sensitivity to early changes in bone remodeling during orthodontic tooth movement.

Moderate dental crowding is a common indication for orthodontic treatment, often requiring space management strategies such as tooth extraction [6]. In such cases, the leveling and alignment phase represents the initial stage of fixed appliance therapy and is characterized by increased biological activity resulting from tooth movement under light and continuous forces [7]. Tooth extraction remains an integral adjunctive procedure in orthodontic treatment, particularly in cases of moderate to severe crowding, as it provides the space required for proper alignment and occlusal correction [8]. However, despite its widespread use, the optimal timing of tooth extraction in relation to the initiation of orthodontic alignment continues to be a subject of ongoing debate [9].

Importantly, this debate extends beyond mechanical treatment planning considerations to encompass relevant biological aspects. Tooth extraction induces localized alveolar bone healing and remodeling processes, which may influence the surrounding periodontal environment and interact with subsequent orthodontic tooth movement. Within this context, numerous studies have investigated alkaline phosphatase activity in gingival crevicular fluid during the leveling and alignment phase or during active orthodontic tooth movement. However, these studies either did not clearly define the timing of tooth extraction relative to the initiation of orthodontic force application, or performed extraction with a sufficiently long interval prior to orthodontic treatment, thereby effectively isolating the early biological effect of extraction from the biological response associated with orthodontic tooth movement [10–13]. Consequently, the biological response related to the temporal overlap between extraction-induced bone remodeling and the initiation of orthodontic force application remains insufficiently characterized, particularly with respect to the interaction between these processes, representing a clinically relevant knowledge gap.

It was hypothesized that different extraction timing protocols would result in distinct patterns of ALP activity in gingival crevicular fluid during the early phase of orthodontic treatment.

Accordingly, this randomized controlled trial aimed to investigate changes in alkaline phosphatase activity in gingival crevicular fluid around maxillary canines under three orthodontic protocols: leveling and alignment alone, extraction alone, and simultaneous extraction with leveling and alignment, to investigate early biochemical differences associated with extraction timing during orthodontic treatment.

## Methods

### Study design and ethical approval

This randomized, controlled, three-arm clinical trial was conducted at the Department of Orthodontics, Faculty of Dentistry, University of Damascus (Damascus, Syria) between 2022 and 2024. The study protocol was approved by the University of Damascus Research Ethics Committee, under approval number DN-160925-H35. The trial was registered in the ISRCTN Registry (ISRCTN17615120) on 30 October 2025. The trial was registered retrospectively due to administrative delays; however, the study protocol and methodology were predefined prior to participant enrollment and were not modified after trial initiation. The full trial record is publicly accessible at https://www.isrctn.com/ISRCTN17615120, Written informed consent was obtained from all participants prior to enrollment and randomization. All procedures complied with the Declaration of Helsinki [14] and were conducted in accordance with the CONSORT 2010 guidelines for randomized clinical trials [15].

Randomization was performed using a computer-generated random sequence with a 1:1:1 allocation ratio. Allocation concealment was ensured using sequentially numbered, sealed, opaque envelopes prepared by an independent researcher not involved in participant recruitment or outcome assessment.

### Sample size calculation

Sample size was determined using G*Power software (version 3.1.9.7; Heinrich-Heine-Universität Düsseldorf, Germany) based on an F-test (one-way ANOVA, fixed effects, omnibus) comparing three independent groups for the primary outcome of interest, for inter-group differences in ALP activity. A two-sided α level of 0.05, a statistical power of 0.95, and an effect size of Cohen’s *f* = 0.55 (derived from the inter-group differences reported by Perinetti et al. [5]) were assumed. This analysis indicated a required total sample size of 55 participants (approximately 19 per group). To ensure adequate power, allow for potential dropouts, and maintain equal group allocation, 60 patients were ultimately recruited (20 per group).

### Participant enrollment

Seventy patients were screened according to predefined eligibility criteria. Diagnostic records included study models, panoramic radiographs, and lateral cephalometric radiographs. Ten patients were excluded (seven did not meet the inclusion criteria, and three declined participation). The remaining 60 eligible participants were enrolled and randomly allocated in equal numbers to the three study groups (*n* = 20 each).

## Eligibility criteria

### Inclusion criteria

The inclusion criteria comprised adults aged 18–25 years; patients whose treatment plan required bilateral extraction of the maxillary first premolars; moderate maxillary crowding based on Little’s Irregularity Index [6]; a normal vertical skeletal pattern (Björk sum between 390° and 402°); complete permanent dentition excluding third molars; and good systemic health. In addition, periodontal health was well controlled throughout the study, with both the Plaque Index and the Gingival Index maintained below 1 during all follow-up visits.

### Exclusion criteria

Systemic diseases affecting bone or periodontal metabolism; previous orthodontic treatment; pregnancy; growth disturbances; severely ectopic anterior teeth preventing alignment; chronic use of medications influencing bone turnover (e.g., corticosteroids, NSAIDs); poor oral hygiene; gingivitis or periodontitis; craniofacial anomalies such as cleft lip and/or palate.

### Randomization and blinding

Participants were randomly allocated to the three groups (*n* = 20 each) using a computer-generated random sequence. Allocation concealment was ensured through the use of sealed, opaque, sequentially numbered envelopes prepared by an independent investigator who was not involved in any clinical or laboratory procedures. Each GCF sample was coded with a unique identifier by an independent coordinator. Laboratory examiners analyzing ALP activity were blinded to group allocation. Blinding of the operator and participants was not feasible due to the nature of the interventions. Baseline comparability among groups in age, sex distribution, growth pattern, and initial crowding was confirmed.

### Clinical procedures

Patients in all groups were treated according to standardized clinical protocols.

#### Group A—Leveling and alignment with delayed extraction

Leveling and alignment were initiated immediately at baseline, while bilateral extraction of the maxillary first premolars was delayed for 6 weeks.

#### Group B—Extraction only

Bilateral extraction of the maxillary first premolars was performed at baseline without placement of orthodontic appliances during the 6-week observation period. Anchorage was maintained using either a transpalatal arch (TPA) or a TPA combined with a Nance appliance, based on individual clinical needs. Following completion of the final gingival crevicular fluid sample collection, orthodontic appliance placement was resumed, and orthodontic treatment was continued routinely according to the planned treatment protocol.

#### Group C—Simultaneous extraction with leveling and alignment

Bilateral extractions were performed first, followed by a waiting period of approximately two hours to ensure hemostasis and blood clot stabilization. Leveling and alignment were then initiated during the same visit.

In Groups A and C, leveling and alignment were performed using 0.022-inch MBT prescription brackets (American Orthodontics, Sheboygan, WI, USA). A predetermined nickel–titanium (NiTi) archwire sequence (American Orthodontics) was followed: 0.014 → 0.016 → 0.016 × 0.016 → 0.016×0.022 → 0.017 × 0.025 in. Each archwire was maintained for an average period of approximately three weeks, with minor variations among patients depending on the individual progress during the leveling and alignment phase, with weekly replacement of elastic modules. Progression to the subsequent archwire occurred only after the preceding wire was fully seated within the brackets with minimal deformation. To complete the alignment phase, a 0.017 × 0.025-in. stainless steel archwire was used for 3 weeks. During the six-week study period, only the initial archwires (0.014-in., 0.016-in., and occasionally 0.016 × 0.016-in. NiTi) were used, depending on the degree of individual alignment progression. The orthodontic protocol was standardized across all participants. Initial alignment was performed using light continuous forces delivered through sequential nickel–titanium archwires. The exact magnitude of force applied to individual teeth, particularly canines, during the leveling and alignment phase could not be precisely quantified. However, consistency was ensured by applying the same archwire sequence and standardized clinical protocol in all patients. All procedures were performed by the same operator to minimize inter-operator variability.

All participants received standardized oral hygiene instructions at the first visit. They were instructed to refrain from eating or drinking for 6 h prior to gingival crevicular fluid collection.

### Gingival crevicular fluid (GCF) sampling protocol

Gingival crevicular fluid (GCF) was collected at seven time points: T0 (baseline), T1 (7 days), T2 (14 days), T3 (21 days), T4 (28 days), T5 (35 days), and T6 (42 days). Samples were obtained simultaneously from the Mesial and Distal sites of the right and left maxillary canines using sterile #30 paper points. Each paper point was gently inserted into the gingival crevice until slight resistance was felt and left in place for 60 s. For each Mesial or Distal site, four paper points were collected (two from the right canine and two from the left canine), with a 60-s interval between insertions to ensure adequate fluid absorption and enhance measurement reliability.

**Fig. 1:**
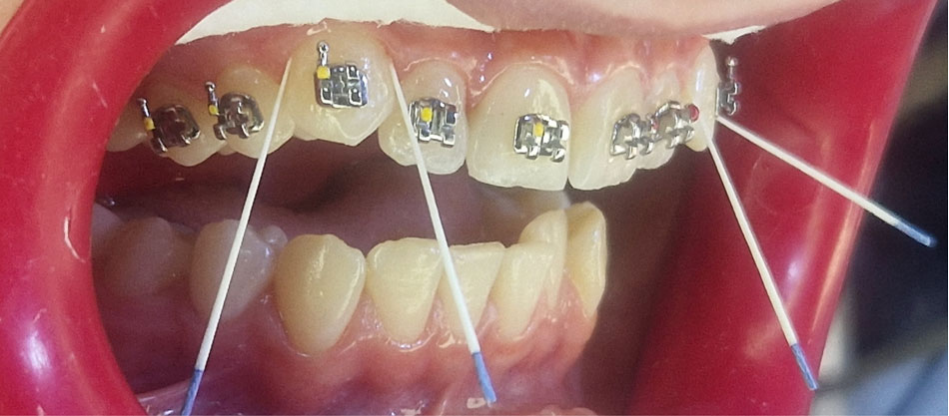
Method of sample collection using paper points.

To standardize sample volume, the same paper point size and insertion duration were used for all subjects at all time points. Each sample was immediately placed into 1.5-mL Eppendorf tubes containing 225 μL of carbonate buffer, kept on ice, and transported promptly to the laboratory (Fig. 1). In total, 840 GCF samples were collected (two sites × seven time points × sixty patients). All samples were coded using unique identification numbers by an independent coordinator, ensuring complete blinding of laboratory investigators. All clinical measurements and GCF sample collections were performed by the same trained examiner to ensure consistency and reduce variability. To enhance sample volume and improve measurement reliability, repeated absorption was performed using multiple paper points at each sampling site. This approach was based on previous evidence indicating that the gingival sulcus replenishes gingival crevicular fluid within a short time after absorption, allowing the collection of newly formed fluid that reflects ongoing biological activity. In addition, repeated sampling has been shown to increase sample volume and reduce variability between measurements. Samples contaminated with blood or saliva during collection were excluded from the analysis to ensure accuracy of the biochemical measurements.

### Laboratory analysis

GCF samples were centrifuged at 2000 × *g* for 5 min (Micro Centaur, UK) to elute the fluid into the buffer. The paper points were then discarded, and 200 μL of the supernatant was analyzed using a spectrophotometric method with a spectrophotometer (Jenway 6330, UK) at a wavelength of 405 nm. Alkaline phosphatase (ALP) activity was expressed in international units per liter (IU/L) according to the manufacturer’s calibration standards. All biochemical analyses were performed by a single blinded examiner to minimize measurement bias.

Analyses were conducted within 24 h of sample processing, while the remaining samples were stored at –70 °C for potential repeat testing. Calibration checks were performed before every analytical session.

### Periodontal health assessment

Periodontal health was assessed at baseline and at each sampling time point to minimize the influence of gingival inflammation on GCF biomarkers. Clinical evaluation included visual inspection to confirm the absence of plaque accumulation and gingival inflammation, as well as the absence of bleeding on probing. In addition, participants were asked about gingival bleeding during tooth brushing, and only those reporting no bleeding were considered to have adequate plaque control. Orthodontic treatment was initiated only after confirming an adequate level of oral hygiene, and oral hygiene instructions were reinforced at each follow-up visit.

No signs of gingival inflammation were observed at any sampling site throughout the study period. Therefore, no samples were excluded due to inflammation. However, samples contaminated with blood or saliva during collection were excluded from the analysis.

### Variables and statistical analysis

Statistical analyses were performed using SPSS software, version 28.0 (IBM Corp., Armonk, NY, USA). The dependent variable was alkaline phosphatase (ALP) activity (IU/L), measured at Mesial and Distal sites across seven time points (T0–T6), while the independent variable was the treatment group.

The primary outcome was the between-group difference in ALP activity across repeated time points (T0–T6). The primary endpoint was defined as the overall longitudinal pattern of ALP activity across the entire follow-up period rather than at a single time point. Secondary outcomes included intragroup temporal changes and site-specific differences between Mesial and Distal measurements. ALP measurements obtained from Mesial and Distal sites were analyzed separately to account for potential site-specific biological differences associated with orthodontic tooth movement.

Data normality was assessed using the Kolmogorov–Smirnov test. Because most ALP measurements did not meet the assumptions of normal distribution and homogeneity of variance, non-parametric tests were primarily applied. Intragroup temporal changes were evaluated using Friedman’s test, followed by Wilcoxon signed-rank post-hoc comparisons when applicable. Intergroup differences at each time point were assessed using the Kruskal–Wallis test with Mann–Whitney U tests for pairwise comparisons with appropriate adjustment for multiple testing (Bonferroni correction when applicable). When both normality and homoscedasticity assumptions were satisfied, one-way ANOVA with Bonferroni-adjusted post-hoc tests was applied. All tests were two-sided, and statistical significance was set at *p* < 0.05.

Although linear mixed-effects models are commonly used for longitudinal data, they were not adopted in this study because key assumptions, including normality of residuals, homogeneity of variance over time, and stable covariance structures, were not consistently met in our dataset. In addition, the relatively small sample size within each group (*n* = 20) and the presence of multiple repeated measurements from two anatomical sites (Mesial and Distal) introduced hierarchical dependencies that could not be modeled reliably without substantial risk of overfitting.

To account for within-subject correlations arising from repeated measurements over time and across anatomical sites, non-parametric repeated-measures tests were used, as they are robust to violations of independence assumptions in small-sample longitudinal data. Effect sizes were estimated using appropriate measures for non-parametric data, and confidence intervals were reported where applicable to support the interpretation of results

## Results

**Fig. 2:**
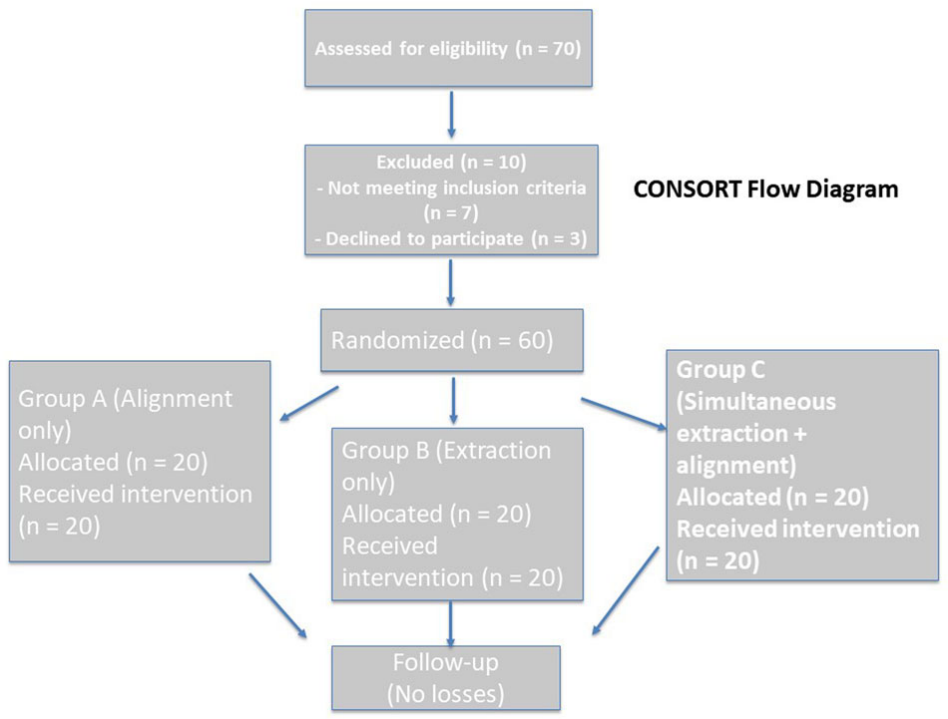
CONSORT flow diagram showing patient enrollment, allocation, follow-up, and analysis.

A total of 70 patients presenting with moderate maxillary crowding and requiring bilateral extraction of the upper first premolars as part of orthodontic treatment were initially screened for eligibility. Ten patients were excluded: seven did not meet the inclusion criteria, and three declined to participate. The remaining 60 eligible participants were enrolled and randomly assigned in equal numbers (*n* = 20 per group) to the three study groups using sealed opaque envelopes to ensure allocation concealment (Fig. 2).

Group A (leveling and alignment with delayed extraction) and Group B (extraction only) had an equal gender distribution (50% male and 50% female), with mean ages of 20.00 ± 1.45 and 21.60 ± 2.64 years, respectively. Group C (simultaneous extraction with leveling and alignment) consisted of 40% males and 60% females, with a mean age of 21.80 ± 1.98 years. There were no statistically significant differences among the groups regarding gender or age distribution, confirming baseline comparability (Table 1).

**Table 1:** The distribution of age and gender among the three study groups.

	Groups		Value	Percentage	*P*-value
Gender	Group A	Male	10	50%	0.522
		female	10	50%	
	Group B	Male	10	50%	
		female	10	50%	
	Group C	Male	8	40%	
		female	12	60%	
Age	Group A, B, C	Minimum	18		0.075
		Maximum	25		
		Mean	21.15 ± 2.30		

Normality testing revealed that most ALP values at both Mesial and Distal sites were not normally distributed, except for Mesial measurements at T3 and Distal measurements at T5 and T6. Consequently, non-parametric statistical tests were primarily applied, while parametric analyses were reserved for normally distributed datasets.

### Inter-group comparisons

No significant differences were detected at baseline, confirming the homogeneity of the three groups prior to intervention. From week 1 onward, inter-group differences became evident.

At Mesial sites, ALP activity in Group B was higher than in Group A at several time points. At week 2, the mean difference (B–A) was 1.42 (95% CI: 0.60–2.24), and at week 3 it remained elevated at 1.42 (95% CI: 0.94–1.90). At later time points, this difference persisted, with values of 1.34 (95% CI: 1.00–1.68) at week 5 and 1.20 (95% CI: 0.87–1.53) at week 6.

Comparisons between Group C and Group A showed smaller differences at early time points, but became more evident at later stages. At week 5, the mean difference (C–A) was 0.50 (95% CI: 0.03–0.97), and at week 6 it was 0.49 (95% CI: 0.02–0.96).

Between Groups B and C, differences varied across time points. At week 3, Group B exceeded Group C with a mean difference of −1.33 (95% CI: −1.71 to −0.95) (C–B), while at weeks 5 and 6, Group B remained higher, with differences of −0.84 (95% CI: −1.19 to −0.49) and −0.71 (95% CI: −1.06 to −0.36), respectively.

At Distal sites, a similar pattern was observed. Early differences were modest; however, from week 2 onward, Group B showed higher ALP activity compared with Group A, with a mean difference of 1.42 (95% CI: 0.60–2.24) at week 2 and 1.42 (95% CI: 0.94–1.90) at week 3. At later time points, differences remained evident, particularly at week 6 (1.20 (95% CI: 0.87–1.53)).

Comparisons between Groups B and C demonstrated that Group B maintained higher values at several time points, particularly at weeks 5 and 6, while differences between Groups A and C remained smaller throughout the observation period.

**Fig. 3:**
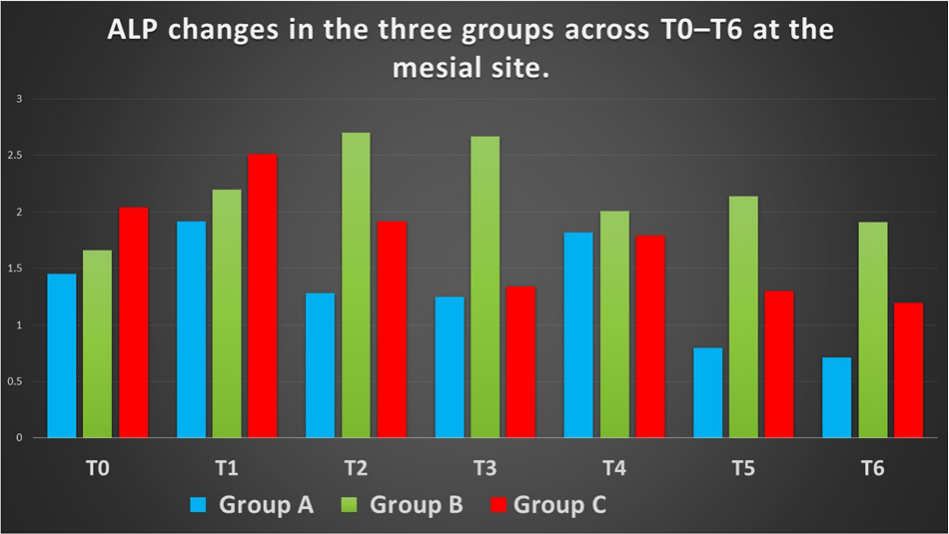
Mean ALP activity (IU/L) across time points in the three groups (Mesial sites).

**Fig. 4:**
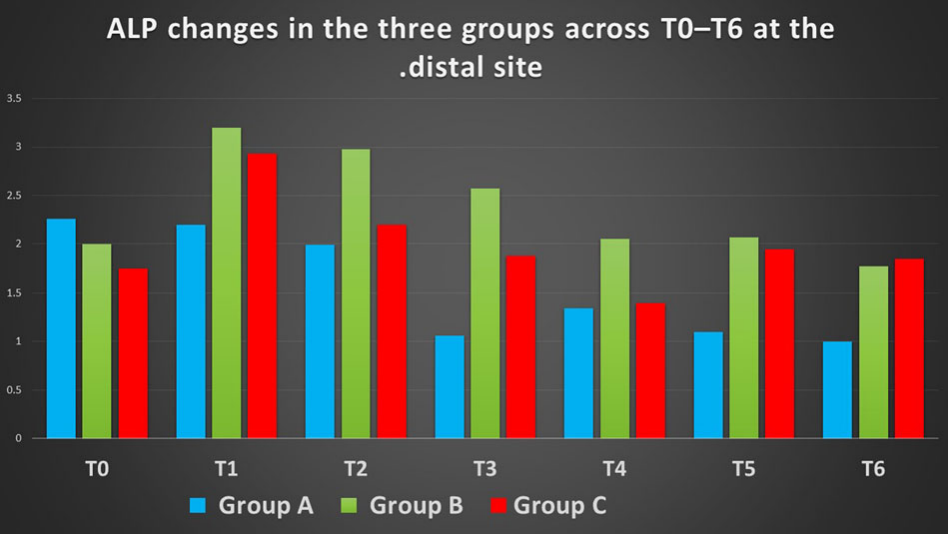
Mean ALP activity (IU/L) across time points in the three groups (distal sites).

These inter-group differences were supported by Kruskal–Wallis and ANOVA analyses, with detailed pairwise comparisons presented in Tables (2–5) and Figs. (3–4).

**Table 2:** Friedman test results for temporal changes in ALP activity at mesial and distal sites within the three groups.

The non-parametric Friedman test																		
	Group A						Group B						Group C					
	Mean				*P*-value		Mean				*P*-value		Mean				*P*-value	
	M	SD	D	SD	M	D	M	SD	D	SD	M	D	M	SD	D	SD	M	D
T0	1.45	0.6	2.26	1.19	0.001*	0.001*	1.66	0.52	2	0.39	0.001*	0.001*	2.04	0.99	1.75	1.11	0.018*	0.201
T1	1.92	1.24	2.2	1.12			2.2	0.34	3.2	0.32			2.51	4.21	2.93	2.97		
T2	1.28	1.01	1.99	1.72			2.7	1.41	2.98	1.01			1.92	1.71	2.2	1.5		
T3	1.25	0.98	1.06	0.74			2.67	0.25	2.57	0.22			1.34	0.75	1.88	1.87		
T4	1.82	1.19	1.34	0.76			2.01	0.37	2.05	0.12			1.79	1.2	1.4	0.78		
T5	0.8	0.69	1.1	0.56			2.14	0.19	2.07	0.32			1.3	0.72	1.95	1.01		
T6	0.71	0.68	1	0.56			1.91	0.17	1.77	0.34			1.2	0.72	1.85	1.01		

*M* mesial, *D* distal, *SD* standard deviation.

*Statistically significant difference (*p* < 0.05).

**Table 3:** Wilcoxon pairwise comparisons of ALP activity across time points at mesial and distal sites within each group.

The non-parametric Wilcoxon test							
	Time intervals	*P*-value					
		Group A		Group B		Group C	
		M	D	M	D	M	D
T0	T1	0.036*	0.678	0.005*	0.001*	0.092	-
	T2	0.574	0.027*	0.001*	0.001*	0.708	-
	T3	0.471	0.011*	0.001*	0.001*	0.012*	-
	T4	0.027*	0.016*	0.049*	0.251	0.525	-
	T5	0.038*	0.002*	0.001*	0.384	0.002*	-
	T6	0.016*	0.001*	0.029*	0.007*	0.001*	-
T1	T2	0.043*	0.247	0.143	0.013*	0.879	-
	T3	0.585	0.001*	0.001*	0.001*	0.881	-
	T4	0.003*	0.001*	0.643	0.001*	0.419	-
	T5	0.001*	0.001*	0.554	0.001*	0.795	-
	T6	0.001*	0.001*	0.010*	0.001*	0.331	-
T2	T3	0.975	0.009*	0.095	0.012*	0.125	-
	T4	0.052	0.246	0.164	0.001*	0.97	-
	T5	0.034*	0.024*	0.012*	0.001*	0.349	-
	T6	0.006*	0.018*	0.001*	0.001*	0.246	-
T3	T4	0.121	0.231	0.001*	0.001*	0.108	-
	T5	0.008*	0.917	0.001*	0.001*	0.527	-
	T6	0.011*	0.646	0.001*	0.001*	0.736	-
T4	T5	0.017*	0.388	0.664	0.776	0.647	-
	T6	0.005*	0.19	0.07	0.001*	0.348	-
T5	T6	0.001*	0.001*	0.001*	0.013*	0.001*	-

*Statistically significant difference (*p* < 0.05).

**Table 4:** Kruskal–Wallis test comparing ALP activity among the three groups at each time point.

Kruskal-Wallis test							
	-	-	Median	Mean	Standard deviation	Test value	*P*-value
T0	Mesial	A	1.25	451	0.6	10.148	120
		B	1.75	1.66	0.52		
		C	1.8	2.04	0.99		
	Distal	A	2.25	2.26	1.19	5.854	0.119
		B	2	2	0.39		
		C	1.5	1.75	1.11		
T1	Mesial	A	1.9	1.92	1.24	13.039	0.005*
		B	2.2	2.2	0.34		
		C	1.25	2.51	4.21		
	Distal	A	2	2.2	1.12	20.464	0.001*
		B	3.25	3.2	0.32		
		C	1.5	2.39	2.97		
T2	Mesial	A	1.2	1.28	1.01	20.371	0.001*
		B	2.25	2.7	1.41		
		C	1.3	1.92	1.71		
	Distal	A	1.6	1.99	1.72	19.049	0.001*
		B	2.75	2.98	1.01		
		C	1.5	2.2	1.5		
T3	Distal	A	0.85	1.06	0.74	33.925	0.001*
		B	2.55	2.57	0.22		
		C	1.4	1.88	1.87		
T4	Mesial	A	1.75	1.82	1.19	6.955	0.073
		B	2.05	2.01	0.37		
		C	1.7	1.79	1.2		
	Distal	A	1.15	1.34	0.76	16.053	0.001*
		B	2	2.05	0.12		
		C	1.4	1.4	0.78		
T5	Mesial	A	0.6	0.8	0.69	37.916	0.001*
		B	2.1	2.14	0.19		
		C	1.15	1.3	0.72		
T6	Mesial	A	0.5	0.71	0.68	32.349	0.001*
		B	1.95	1.91	0.17		
		C	1.05	1.2	0.72		

*Statistically significant difference (*p* < 0.05).

**Table 5:** Mann–Whitney pairwise group comparisons of ALP activity at each time point.

The non-parametric Mann–Whitney U test for pairwise comparisons				
		Compared group	Test value	*P*-value
T1 Mesial	Group A	Group B	124	0.039*
		Group C	146	0.143
	Group B	Group C	86	0.002*
T1 distal	Group A	Group B	48	0.001*
		Group C	198	0.957
	Group B	Group C	160	0.278
T2 Mesial	Group A	Group B	68	0.001*
		Group C	154	0.211
	Group B	Group C	108	0.013*
T2 distal	Group A	Group B	96	0.005*
		Group C	184	0.664
	Group B	Group C	112	0.017*
T3 distal	Group A	Group B	94	0.004*
		Group C	18	0.001*
	Group B	Group C	142	0.001*
T4 distal	Group A	Group B	94	0.004*
		Group C	172	0.448
	Group B	Group C	96	0.005*
T5 Mesial	Group A	Group B	34	0.001*
		Group C	114	0.020*
	Group B	Group C	42	0.001*
T6 Mesial	Group A	Group B	40	0.001*
		Group C	114	0.020*
	Group B	Group C	60	0.001*

*Statistically significant difference (*p* < 0.05).

### Intra-group findings

Distinct temporal patterns of ALP activity were observed within each treatment group.

In Group A (leveling and alignment with delayed extraction), Mesial ALP activity increased from baseline (T0: 1.45 IU/L; 95% CI: 1.01–1.57) to a peak at week 1 (T1: 1.92 IU/L; 95% CI: 1.34–2.50), followed by a progressive decline over time, reaching its lowest level at week 6 (T6: 0.71 IU/L; 95% CI: 0.39–1.03).

In contrast, Distal ALP levels were initially higher at baseline (T0: 2.26 IU/L; 95% CI: 1.70–2.82) and demonstrated a gradual decrease throughout the observation period, with more pronounced reductions observed from week 3 onward (T3: 1.06 IU/L; 95% CI: 0.71–1.41), reaching 1.00 IU/L (95% CI: 0.74–1.26) at week 6.

In Group B (extraction only), Mesial ALP increased from 1.66 IU/L (95% CI: 1.42–1.91) at baseline to a peak at week 2 (2.70 IU/L; 95% CI: 2.04–3.36), followed by a gradual decline to 1.91 IU/L (95% CI: 1.83–1.99) at week 6. In contrast, Distal ALP showed an early sharp increase from 2.00 IU/L (95% CI: 1.82–2.19) at baseline to 3.20 IU/L (95% CI: 3.05–3.35) at week 1, then decreased over time to 1.77 IU/L (95% CI: 1.61–1.93) at week 6.

In Group C (simultaneous extraction with leveling and alignment), Mesial ALP values at baseline were 2.04 (95% CI: 1.57–2.50). At week 1, values were 2.51 (95% CI: 0.54–4.48), followed by 1.92 (95% CI: 1.12–2.72) at week 2 and 1.34 (95% CI: 0.99–1.69) at week 3. At week 6, Mesial ALP decreased to 1.20 (95% CI: 0.86–1.54).

Distally, baseline ALP values were 1.75 (95% CI: 1.23–2.27), increasing to 2.93 (95% CI: 1.54–4.32) at week 1. Subsequent values were 2.20 (95% CI: 1.35–3.05) at week 2 and 1.88 (95% CI: 1.00–2.76) at week 3. At week 6, Distal ALP values were 1.85 (95% CI: 1.38–2.32).

**Fig. 5:**
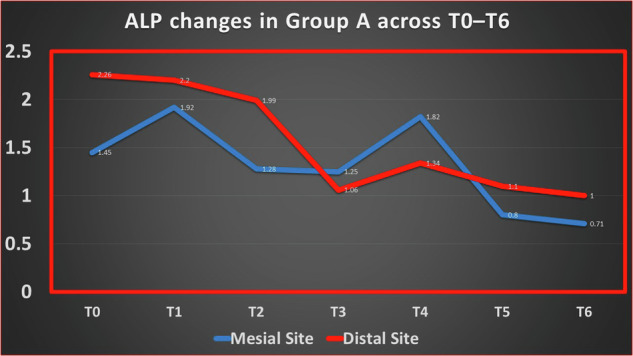
Changes in ALP activity (IU/L) across time points in Group A.

**Fig. 6:**
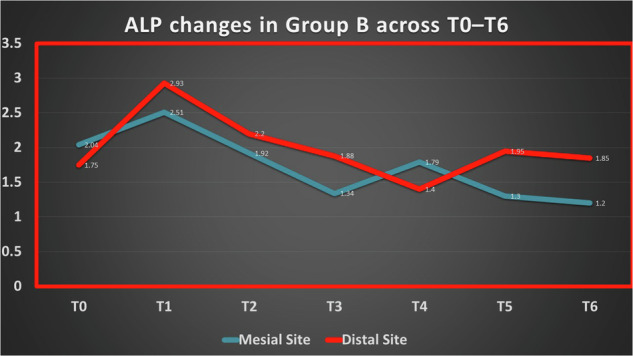
Changes in ALP activity (IU/L) across time points in Group B.

**Fig. 7:**
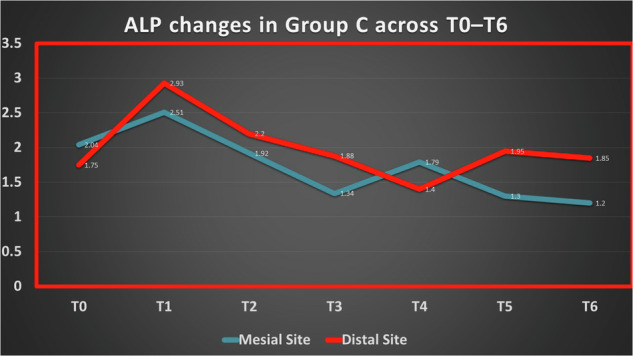
Changes in ALP activity (IU/L) across time points in Group C.

Detailed statistical results for temporal changes are presented in Tables (6a–c and 7a–c) and Figs. (5–7).

**Table 6:** One-way ANOVA results for intergroup differences in ALP activity at normally distributed time points.

One-Way ANOVA test							
	-	-	Median	Mean	Standard deviation	Test value	*P*-value
T3	Mesial	A	1.2	1.25	0.98		0.001^*^
		B	2.75	2.67	0.25		
		C	1.1	1.34	0.75		
T5	Distal	A	1.1	1.1	0.56		0.001*
		B	2	2.07	0.32		
		C	2.1	1.95	1.01		
T6	Distal	A	1	1	0.56		0.001*
		B	1.7	1.77	0.34		
		C	2	1.85	1.01		

*Statistically significant difference (*p *< 0.05).

**Table 7:** Bonferroni post-hoc comparisons of ALP activity among groups at normally distributed time points.

Bonferroni correction for pairwise comparisons			
	-	Compared group	*P*-value
Time point:T3 mesial	Group A	Group B	0.001*
		Group C	0.664
	Group B	Group C	0.001*
Time point:T5 distal	Group A	Group B	0.001*
		Group C	0.001*
	Group B	Group C	0.55
Time point:T6 distal	Group A	Group B	0.001*
		Group C	0.002*
	Group B	Group C	1

*Statistically significant difference (*p* < 0.05).

## Discussion

This randomized clinical trial examined how different extraction protocols influence periodontal biological activity during the early stages of orthodontic treatment, focusing on alkaline phosphatase (ALP) as a marker of bone formation.

The importance of the present study aim arises from the fact that tooth extraction is widely employed as part of orthodontic treatment protocols, particularly in patients with moderate to severe crowding. Despite its frequent use, available clinical evidence remains insufficient to define the optimal timing of tooth extraction for individual orthodontic cases, and there is a notable lack of clinical studies that have directly addressed the early biological effects of tooth extraction on periodontal tissues [7, 16]. In light of this knowledge gap, the present study aimed to demonstrate and monitor the subclinical biological effects induced by tooth extraction, with particular emphasis on its potential to activate the regional acceleratory phenomenon (RAP), as described by Frost [17]. This biological response was investigated both when extraction was performed alone and when it was combined with orthodontic force application, considering the possible interaction between extraction-related bone healing and orthodontic tooth movement. Furthermore, this study sought to explore whether early biochemical responses associated with different extraction protocols, without direct assessment of clinical tooth movement by analyzing changes in alkaline phosphatase (ALP) activity in gingival crevicular fluid as a biomarker of localized periodontal and bone metabolic activity.

Alkaline phosphatase (ALP) was selected as the primary biomarker in the present study due to its well-established and close association with bone formation and remodeling processes, making it one of the most extensively investigated enzymes in orthodontic research [12, 18]. ALP plays a key role in early osteoblastic differentiation and the initiation of matrix mineralization, rendering it a sensitive indicator of localized biological changes accompanying orthodontic tooth movement [19].

Beyond its biological relevance, ALP offers several practical advantages for clinical research, as its analytical methods are relatively simple, cost-effective, and highly reproducible [5, 12]. Furthermore, previous studies have demonstrated that ALP exhibits high sensitivity even in small-volume biological samples, such as gingival crevicular fluid, which supports its reliability as a biomarker of periodontal and alveolar bone metabolic activity during orthodontic treatment [13, 18, 19].

The study sample was restricted to young adults aged 18–25 years, as skeletal growth at this age is completed or nearly completed, thereby minimizing the confounding effect of growth on bone metabolism and alkaline phosphatase activity [1, 2]. Excluding the influence of growth is particularly important in biomarker-based studies to ensure that the measured changes reflect biological responses to therapeutic stimuli rather than physiological developmental variations.

Conversely, older age groups are associated with a higher prevalence of systemic diseases or periodontal alterations that may directly or indirectly influence enzymatic activity, particularly ALP levels [7]. Accordingly, this age selection enhanced sample homogeneity and ensured that the observed changes in ALP activity primarily reflected treatment-related biological responses.

This study adopted Little’s Irregularity Index to define moderate crowding within the range of 4–6 mm, as this represents a borderline category that may be managed using either extraction or non-extraction protocols depending on individual case characteristics. This level of crowding is particularly suitable for investigating the biological effects of tooth extraction and its timing, while allowing extraction to be delayed without compromising orthodontic treatment objectives. Severe crowding, on the other hand, is typically associated with early extraction protocols, more complex tooth movements, and greater difficulty in maintaining adequate oral hygiene, all of which may increase biological variability and reduce sample homogeneity. Conversely, mild crowding is usually managed without extraction [7]. Therefore, selecting moderate crowding ensured an optimal balance between clinical relevance and biological consistency.

Pregnant patients were excluded due to the well-documented physiological elevation of alkaline phosphatase activity during pregnancy, which is independent of local orthodontic or inflammatory stimuli [20]. In addition, patients with systemic diseases known to affect ALP activity such as liver and kidney disorders, endocrine abnormalities, and diabetes mellitus were excluded, given their direct influence on bone and enzymatic metabolism [21, 22]. Previous studies have emphasized the necessity of excluding any condition that may independently alter ALP levels, in order to preserve the biological validity of biomarker-based investigations [23] This approach minimized confounding factors and ensured that the measured changes in ALP activity reflected localized responses to orthodontic forces or tooth extraction alone. [24]

The present study included only patients with a normal vertical growth pattern based on Björk’s analysis to avoid functional and inflammatory variability associated with other growth patterns. Patients with a horizontal growth pattern are often treated using non-extraction protocols, which could limit intergroup comparability. Conversely, a vertical growth pattern is frequently associated with functional characteristics such as mouth breathing, open bite, and lip incompetence, all of which increase susceptibility to gingival inflammation and may influence biomarker levels in gingival crevicular fluid [7, 25]. Therefore, restricting the sample to individuals with a normal growth pattern enhanced sample homogeneity and improved the accuracy of interpreting biological changes in ALP activity.

The study required patients to achieve an adequate level of oral hygiene prior to orthodontic bracket placement, given the high sensitivity of gingival biomarkers to inflammatory changes. Oral health status was assessed using simple yet effective clinical criteria, including visual examination to confirm the absence of plaque accumulation on teeth and gingiva and the absence of clinical signs of gingival inflammation. In addition, a verbal assessment was conducted by inquiring about the presence of gingival bleeding after tooth brushing, with a negative response considered indicative of effective plaque control. Orthodontic treatment was initiated only when both criteria were fulfilled, and oral hygiene instructions were reinforced at every follow-up visit [26].

Strict oral hygiene control was essential in the present study because gingival crevicular fluid represents a highly sensitive medium that responds rapidly to inflammatory stimuli. The placement of orthodontic appliances alone may increase ALP activity due to plaque accumulation, independent of orthodontic force application [5]. Therefore, rigorous oral hygiene control ensured that the observed changes in ALP activity reflected true biological responses to orthodontic forces or tooth extraction rather than secondary inflammatory effects.

This study adopted weekly replacement of elastic modules, deviating from routine orthodontic practice, in order to control a major confounding factor that could influence the biological response of periodontal tissues during orthodontic treatment. Elastic modules are known to exhibit a high tendency for plaque accumulation and to lose their mechanical properties shortly after placement, which may amplify inflammatory and enzymatic responses independently of the orthodontic force itself. Previous studies have demonstrated that conventional brackets combined with elastic ligation are associated with increased microbial colonization and elevated levels of inflammatory mediators and bone remodeling enzymes compared with self-ligating brackets [26]. Accordingly, the weekly replacement of elastic modules in the present study was a deliberate methodological decision aimed at minimizing cumulative plaque-related effects and maintaining more stable mechanical and biological conditions throughout the measurement periods. This approach allowed for a more accurate reflection of the true biological response of the supporting tissues to orthodontic forces and enhanced the reliability of the findings obtained during the leveling and alignment phase.

Maxillary canines were selected as measurement sites due to their relatively large root surface area and pronounced cellular activity during orthodontic force application, making them suitable teeth for monitoring biological changes associated with tooth movement. In addition, canines are located in close proximity to extraction sites in extraction-based treatment protocols, allowing assessment of localized biological effects related to both tooth extraction and orthodontic movement. Furthermore, canines are among the most extensively studied teeth in orthodontic literature, which facilitates comparison with previous studies and enhances the scientific relevance of the present findings [18, 23, 27].

This study collected gingival crevicular fluid samples from both the right and left sides to minimize individual side-related variability and to control for potential bias resulting from asymmetrical functional or anatomical differences between the two sides of the dental arch. This approach contributes to a more accurate representation of the overall biological response of the supporting tissues during orthodontic treatment and enhances the reliability and generalizability of the results.

In addition, both Mesial and Distal sites were selected for sample collection, as orthodontic tooth movement is associated with different patterns of tension and compression on opposite sides of the tooth, which may be reflected in localized variations in enzymatic and inflammatory activity. This distinction allows for the detection of subtle site-specific biological differences and facilitates a more precise interpretation of biological changes related to the mechanisms of orthodontic tooth movement. It is noteworthy that most previous studies did not explicitly specify the sampling site, as observed in the study by Abdullah and Rohaya [10], underscoring the methodological relevance of this approach.

This study employed #30 paper points for gingival crevicular fluid collection, as they represent a simple, cost-effective method that does not require advanced technical skills and allows repeated sampling without causing significant gingival irritation. This technique was successfully used by Abdullah and Rohaya in their investigation of ALP activity during the leveling and alignment phase [10]. Furthermore, Kapoor (2019) confirmed that paper points are among the acceptable and effective tools for GCF collection in clinical studies, particularly when assessing enzymatic and biochemical markers [18].

The sampling protocol adopted in this study was based on three consecutive absorption periods of 60 seconds each (60 + 60 seconds), following the recommendations reported in the review by Kapoor (2019), which indicated that 60 seconds represents the optimal duration for collecting an adequate volume of GCF without inducing gingival irritation [18]. It has also been demonstrated that the gingival sulcus replenishes GCF within 30–90 seconds after absorption, allowing collection of newly formed fluid that reflects real-time cellular activity, as described by Lamster [28]. Chapple and Genco further reported that the use of two or more paper points may be necessary to obtain a biologically active fraction of GCF, particularly when enzymatic activity is being evaluated [22]. In addition, Kapoor et al. showed that repeated sampling increases sample volume, improves measurement sensitivity, and reduces variability between readings [18]. Samples contaminated with blood or saliva were therefore excluded to ensure measurement accuracy.

A wavelength of 405 nm is considered a standard for measuring alkaline phosphatase activity in small-volume biological samples, as reported in previous studies [5, 27]. This wavelength allows stable and reliable spectrophotometric readings of the chromogenic reaction products, while offering simplicity and rapid execution compared with immunoassay-based techniques such as ELISA, making it well suited for clinical studies involving repeated measurements and large sample numbers.

A six-week follow-up period with seven consecutive weekly time points (T0–T6) was adopted in this study based on the description provided by Kumar et al. of the ALP activity curve following corticotomy, which demonstrated a sustained increase in ALP levels for up to six weeks [29, 30]. Accordingly, this time frame represents an optimal window for investigating the induction of the regional acceleratory phenomenon (RAP), whether triggered by tooth extraction, orthodontic leveling and alignment, or the combination of both.

The Mesial surface of the maxillary canine in Group A showed an initial increase in ALP activity at T1, followed by a progressive decline toward T6, with a secondary elevation at T4. This pattern may be explained by the biomechanical characteristics of early alignment in non-extraction cases, where multidirectional tooth movements generate alternating pressure and tension zones. As ALP reflects bone formation in tension areas, the predominance of pressure on the Mesial surface may account for the overall reduction in enzymatic activity [1, 2, 7]. The transient increase at T4 may be related to changes in force direction during alignment, allowing temporary re-establishment of tension zones [7, 31].

These findings are consistent with previous reports describing variable enzymatic responses during early orthodontic tooth movement (Perinetti et al. [5]; Krishnan and Meikle [1, 2]), while differing from studies reporting no significant biological response (Abdullah and Rohaya [10]).

On the Distal surface, a progressive reduction in ALP activity was observed, which may reflect sustained pressure conditions generated by rotational and tipping corrections during alignment, as reported in previous studies [1, 2, 19].

In Group B, both Mesial and Distal surfaces demonstrated a pronounced increase in ALP activity during the early time points, followed by a gradual decline toward baseline by week six. This pattern reflects the biological response to tooth extraction and may be consistent with features of the regional acceleratory phenomenon (RAP) as described by Frost [17].

The early elevation in ALP activity may correspond to the inflammatory and reparative phases of alveolar healing, while the subsequent decline may indicate progression toward a remodeling phase [29]. The Distal surface, being closer to the extraction site, showed a more pronounced early increase, consistent with localized biological activation following extraction.

In Group C, the Mesial surface exhibited a modest increase in ALP activity followed by a gradual decline, suggesting a less stable enzymatic response compared with extraction alone. This may be attributed to the interaction between extraction-related biological responses and early alignment forces, which generate variable biomechanical conditions [1, 2, 7].

In contrast, the Distal surface showed a marked early increase in ALP activity, followed by a progressive decline toward baseline. This response may reflect the combined influence of extraction-related biological activity and orthodontic force application in areas adjacent to the extraction site.

These findings differ from those reported by Farahani et al. [27], who observed limited enzymatic changes during alignment following extraction, possibly due to differences in extraction timing and study design.

Group B (extraction only) showed the highest ALP activity across most time points and sites, with only two exception, followed by Group C and then Group A. This finding suggests that tooth extraction is the primary factor driving biological activation on both Mesial and Distal surfaces during the first six weeks.

Group C exceeded Group B at only two locations: T1 on the Mesial surface and T6 on the Distal surface. The early superiority at T1 may be explained by the combined effect of extraction and orthodontic tooth movement, producing the highest biological peak in the study sample. The later difference at T6 may reflect the residual effect of extraction combined with biological variability associated with orthodontic alignment, which can induce transient enzymatic elevations at any stage of leveling.

In contrast, Group A (leveling and alignment only) remained the least biologically active, as orthodontic tooth movement during the leveling and alignment phase is inherently irregular and poorly controlled, failing to establish stable and well-defined pressure and tension zones.

Accordingly, our findings are consistent with previous reports:

Perinetti et al. [5] demonstrated that early alignment forces generate a weak, intermittent, and unstable enzymatic response, significantly lower than that induced by effective orthodontic tooth movement.

Insoft et al. [4] reported that early enzymatic elevations during alignment are limited and transient and do not approach the response associated with true bone injury.

Farahani et al. [27] showed that leveling alone does not produce a sustained or pronounced enzymatic curve, but rather mild fluctuations related to light, non-directed forces, explaining why Group A remained the least active.

Finally, agreement was found with Kumar et al. [29], who demonstrated that surgical interventions induce a stronger enzymatic response than orthodontic tooth movement, as they create clearer and more stable tension and pressure zones compared with the variable and irregular pattern observed during leveling and alignment.

The study offers a novel perspective by directly comparing extraction alone, alignment alone, and their simultaneous application—an approach not previously reported in the literature on GCF biomarkers. This design allowed a clearer understanding of how healing dynamics and mechanical loading overlap during the early stages of orthodontic treatment.

From a clinical perspective, the observed biochemical variations should be interpreted with caution. Although differences in ALP activity reflect changes in early biological responses, they do not necessarily indicate a clinically meaningful acceleration of tooth movement, as actual tooth displacement was not measured in this study.

Several limitations should be acknowledged. The study focused solely on ALP as a marker of bone formation and did not assess complementary resorptive enzymes such as acid phosphatase or TRAP, limiting the ability to characterize the full remodeling cycle. The observation period was restricted to the alignment phase, and actual tooth movement was not quantified, which limits the ability to correlate biochemical activity with mechanical outcomes. In addition, the relatively short follow-up duration (six weeks) restricts the interpretation of longer-term biological responses. The potential influence of sex on ALP activity was not analyzed separately, as the study was not powered to detect sex-based subgroup differences. Furthermore, the presence of multiple comparisons across different time points and sampling sites may increase the risk of type I error despite the application of statistical adjustments.

Future research should integrate additional biomarkers, extend observations into space-closure phases, and directly measure tooth movement to better clarify the biological and clinical implications of extraction timing. Future studies with larger sample sizes should consider sex-based analyses to further explore potential biological variations.

## Conclusions

Within the limitations of this study, the following conclusions can be drawn:

Tooth extraction was associated with increased ALP activity in gingival crevicular fluid during the early phase of orthodontic treatment compared with leveling and alignment alone.

Simultaneous extraction with leveling and alignment resulted in intermediate ALP responses.

These findings suggest that extraction timing modulates early biochemical activity in periodontal tissues.

## Supplementary information

Supplementary Tables

CONSORT-2010-Checklist

## Data Availability

Upon reasonable request, the corresponding author will provide the datasets used and/or analyzed during the current study.
