# Supplementary material for: Alkaline phosphatase activity in gingival crevicular fluid during orthodontic treatment with different extraction protocols for maxillary canines: a randomized controlled trial
Source: BDJ Open. 2026 May 2;12:45. doi: 10.1038/s41405-026-00425-0 (PMC13135509; doi:10.1038/s41405-026-00425-0)
Supplement: Supplementary file 1 — Supplementary Tables [file 41405_2026_425_MOESM1_ESM.docx]

**Supplementary Tables**

**Supplementary Table S1 Wilcoxon pairwise comparisons of ALP activity across time points at mesial and distal sites within each group.**

| **the non-parametric Wilcoxon test** | | | | | | | |
| --- | --- | --- | --- | --- | --- | --- | --- |
| **P-value** | | | | | | **Time**  **intervals** |  |
| **Group C** | | **Group B** | | **Group A** | |  |  |
| D | M | D | M | D | M |  |  |
| - | .092 | .001* | .005* | .678 | .036* | T1 | **T0** |
| - | .708 | .001* | .001* | .027* | .574 | T2 |  |
| - | .012* | .001* | .001* | .011* | ,471 | T3 |  |
| - | .525 | .251 | .049* | .016* | .027* | T4 |  |
| - | .002* | .384 | .001* | .002* | .038* | T5 |  |
| - | .001* | .007* | .029* | .001* | .016* | T6 |  |
| - | .879 | .013* | .143 | .247 | .043* | T2 | **T1** |
| - | .881 | .001* | .001* | .001* | .585 | T3 |  |
| - | .419 | .001* | .643 | .001* | .003* | T4 |  |
| - | .795 | .001* | .554 | .001* | .001* | T5 |  |
| - | .331 | .001* | .010* | .001* | .001* | T6 |  |
| - | .125 | .012* | .095 | .009* | .975 | T3 | **T2** |
| - | .970 | .001* | .164 | .246 | .052 | T4 |  |
| - | .349 | .001* | .012* | .024* | .034* | T5 |  |
| - | .246 | .001* | .001* | .018* | .006* | T6 |  |
| - | .108 | .001* | .001* | .231 | .121 | T4 | **T3** |
| - | .527 | .001* | .001* | .917 | .008* | T5 |  |
| - | .736 | .001* | .001* | .646 | .011* | T6 |  |
| - | .647 | .776 | .664 | .388 | .017* | T5 | **T4** |
| - | .348 | .001* | .070 | .190 | .005* | T6 |  |
| - | .001* | .013* | .001* | .001* | .001* | T6 | **T5** |
| *Statistically significant difference (p < 0.05) | | | | | | | |

**Supplementary Table S2 Mean ALP activity (IU/L) with 95% confidence intervals in Group A.**

| **Mean ALP activity (IU/L) with 95% confidence intervals in Group A** | | | | |
| --- | --- | --- | --- | --- |
| **Time** | **Mesial** | | **Distal** | |
|  | **Mean** | **(95% CI)** | **Mean** | **(95% CI)** |
| **T0** | 1.45 | 1.01–1.57 | 2.26 | 1.70–2.82 |
| **T1** | 1.92 | 1.34–2.50 | 2.20 | 1.67–2.73 |
| **T2** | 1.28 | 0.80–1.76 | 1.99 | 1.18–2.80 |
| **T3** | 1.25 | 0.79–1.71 | 1.06 | 0.71–1.41 |
| **T4** | 1.82 | 1.26–2.38 | 1.34 | 0.98–1.70 |
| **T5** | 0.80 | 0.48–1.12 | 1.10 | 0.84–1.36 |
| **T6** | 0.71 | 0.39–1.03 | 1.00 | 0.74–1.26 |

**Supplementary Table S3 Mean ALP activity (IU/L) with 95% confidence intervals in Group B.**

| **Mean ALP activity (IU/L) with 95% confidence intervals in Group B** | | | | |
| --- | --- | --- | --- | --- |
| **Time** | **Mesial** | | **Distal** | |
|  | **Mean** | **(95% CI)** | **Mean** | **(95% CI)** |
| **T0** | 1.66 | (1.42–1.91) | 2.00 | (1.82–2.19) |
| **T1** | 2.20 | (2.04–2.36) | 3.20 | (3.05–3.35) |
| **T2** | 2.70 | (2.04–3.36) | 2.98 | (2.51–3.45) |
| **T3** | 2.67 | (2.55–2.79) | 2.57 | (2.47–2.68) |
| **T4** | 2.01 | (1.84–2.18) | 2.05 | (1.99–2.11) |
| **T5** | 2.14 | (2.05–2.23) | 2.07 | (1.92–2.22) |
| **T6** | 1.91 | (1.83–1.99) | 1.77 | (1.61–1.93) |

**Supplementary Table S4 Mean ALP activity (IU/L) with 95% confidence intervals in Group C.**

| **Mean ALP activity (IU/L) with 95% confidence intervals in Group C** | | | | |
| --- | --- | --- | --- | --- |
| **Time** | **Mesial** | | **Distal** | |
|  | **Mean** | **(95% CI)** | **Mean** | **(95% CI)** |
| **T0** | 2.04 | 1.58 – 2.50 | 1.75 | 1.23 – 2.27 |
| **T1** | 2.51 | 0.54 – 4.48 | 2.93 | 1.54 – 4.32 |
| **T2** | 1.92 | 1.12 – 2.72 | 2.20 | 1.35 – 3.05 |
| **T3** | 1.34 | 0.99 – 1.69 | 1.88 | 1.00 – 2.76 |
| **T4** | 1.79 | 1.23 – 2.35 | 1.40 | 1.03 – 1.77 |
| **T5** | 1.30 | 0.96 – 1.64 | 1.95 | 1.48 – 2.42 |
| **T6** | 1.20 | 0.86 – 1.54 | 1.85 | 1.38 – 2.32 |

**Supplementary Table S5 pairwise mean differences and 95% confidence intervals in Mesial values**

| **pairwise mean differences and 95% confidence intervals in Mesial values** | | | | | | |
| --- | --- | --- | --- | --- | --- | --- |
| **Time** | **A-B Mean Diff** | **A-B 95% CI** | **A-C Mean Diff** | **A-C 95% CI** | **B-C Mean Diff** | **B-C 95% CI** |
| **T0** | -0.37 | -0.73 to -0.01 | -0.75 | -1.28 to -0.22 | -0.38 | -0.89 to 0.13 |
| **T1** | -0.28 | -0.88 to 0.32 | -0.59 | -2.63 to 1.45 | -0.31 | -2.29 to 1.67 |
| **T2** | -1.42 | -2.21 to -0.63 | -0.64 | -1.55 to 0.27 | 0.78 | -0.23 to 1.79 |
| **T3** | -1.42 | -1.89 to -0.95 | -0.09 | -0.65 to 0.47 | 1.33 | 0.96 to 1.70 |
| **T4** | -0.19 | -0.77 to 0.39 | 0.03 | -0.74 to 0.80 | 0.22 | -0.36 to 0.80 |
| **T5** | -1.34 | -1.67 to -1.01 | -0.50 | -0.96 to -0.04 | 0.84 | 0.49 to 1.19 |
| **T6** | -1.20 | -1.53 to -0.87 | -0.49 | -0.94 to -0.04 | 0.71 | 0.36 to 1.06 |

**Supplementary Table S6 pairwise mean differences and 95% confidence intervals in Distal values**

| **pairwise mean differences and 95% confidence intervals in Distal values** | | | | | | |
| --- | --- | --- | --- | --- | --- | --- |
| **Time** | **A-B Mean Diff** | **A-B 95% CI** | **A-C Mean Diff** | **A-C 95% CI** | **B-C Mean Diff** | **B-C 95% CI** |
| **T0** | 0.26 | -0.32 to 0.84 | 0.51 | -0.23 to 1.25 | 0.25 | -0.29 to 0.79 |
| **T1** | -1.00 | -1.54 to -0.46 | -0.73 | -2.19 to 0.73 | 0.27 | -1.13 to 1.67 |
| **T2** | -0.99 | -1.90 to -0.08 | -0.21 | -1.34 to 0.92 | 0.78 | -0.17 to 1.73 |
| **T3** | -1.51 | -1.87 to -1.15 | -0.82 | -1.75 to 0.11 | 0.69 | -0.19 to 1.57 |
| **T4** | -0.71 | -1.07 to -0.35 | -0.06 | -0.56 to 0.44 | 0.65 | 0.28 to 1.02 |
| **T5** | -1.34 | -1.67 to -1.01 | -0.50 | -0.96 to -0.04 | 0.84 | 0.49 to 1.19 |
| **T6** | -0.77 | -1.07 to -0.47 | -0.85 | -1.38 to -0.32 | -0.08 | -0.57 to 0.41 |

Note: These tables provide detailed statistical results, including intragroup comparisons (Wilcoxon test), descriptive statistics (means and 95% confidence intervals), and pairwise intergroup comparisons (mean differences with 95% confidence intervals), supporting the findings presented in the main manuscript.
